# Determinants of HIV infection among children born to mothers on prevention of mother to child transmission program of HIV in Addis Ababa, Ethiopia: a case control study

**DOI:** 10.1186/s12879-018-3217-3

**Published:** 2018-07-13

**Authors:** Girma Alemayehu Beyene, Lelisa Sena Dadi, Solomon Berhanu Mogas

**Affiliations:** 1 Department of Public Health, College of Health Science and Medicine, Wolkite University, Wolkite, Ethiopia; 2 Department of Epidemiology, Institute of Health, Jimma University, Jimma, Ethiopia

**Keywords:** MTCT, HIV, Option B +, Pediatric HIV, Addis Ababa

## Abstract

**Background:**

Despite wide spread use of Antiretroviral Therapy (ART) by pregnant women living with Human Immunodeficiency Virus (HIV), the transmission rate is still higher by 18% after breastfeeding ends. The aim of this study was to identify factors affecting mother-to-child HIV transmission.

**Methods:**

Unmatched case–control study was conducted in Addis Ababa, from April to May, 2017. A case was HIV positive mother who had been on PMTCT program with her child confirmed HIV positive at or before 24 months and control was HIV positive mother who had been on PMTCT program with her child tested definitive HIV negative at 24 months. Accordingly, 44 cases were identified and for each case four controls with the nearest date of birth to the cases were selected from same health facilities. Primary data collected from the mothers were supplemented by record reviews and entered to Epidata version 3.1 and analyzed using SPSS version 22. Multivariate logistic regression was fitted to identify factors independently associated with mother-to-child HIV transmission.

**Results:**

Lack of participation in mother-to-mother support program (AOR: 5.1; 95% CI: 1.4, 18.1), low partner involvement (AOR: 6.9; 95% CI: 1.4, 13.4), poor ART adherence (AOR:3.1; 95% CI: 1.3, 7.5), positive syphilis test results (AOR: 3.2; 95% CI: 1.2, 8.6), maternal malnutrition (AOR: 3.1; 95% CI: 1.4, 6.8), unplanned pregnancy (AOR: 10.3; 95% CI: 3.9, 27.2), home delivery (AOR: 5.3; 95% CI: 1.4, 19.4) and mixed feeding of the child during first six months of life (AOR: 12.5; 95% CI: 2.9, 52.7) were significantly associated with MTCT of HIV.

**Conclusions:**

Mother-to-mother support, male partner involvement in PMTCT of HIV, strengthening antenatal care, counseling mothers on appropriate infant feeding options are important to reduce mother –to- child transmission of HIV.

**Electronic supplementary material:**

The online version of this article (10.1186/s12879-018-3217-3) contains supplementary material, which is available to authorized users.

## Background

Mother-to-child transmission (MTCT) of HIV occurs when HIV positive woman passes the virus to her baby during pregnancy, childbirth or breastfeeding. It accounts for more than 90% of HIV infections in infants and young children and over 10 % of global HIV infections [1, 2]. The use of Antiretroviral (ARV) drugs is one of the interventions available to prevent transmission of HIV from mother to child during pregnancy, labor and delivery and breastfeeding [3]. ARV prophylaxis, elective caesarean section (CS) and avoidance of breastfeeding reduced risk of MTCT to less than 2 % [4].

More than 90% of world’s HIV-infected children are in sub-Saharan Africa (SSA) [5, 6]. Despite 50% decline in new HIV infections among children since 2010, 150,000 children became newly infected with HIV in 2015 and 56,000 of them were from eastern and southern Africa [7, 8]. Every year, 110,000 children are still newly infected with HIV in the 21 Global Plan priority countries in SSA, including Ethiopia. More than half of new pediatric HIV infections occur during the breastfeeding period and most are infected through vertical transmission [9].

Since 2012, World Health Organization (WHO) recommended Option B+ as an intervention for PMTCT, replacing Cluster of differentiation (CD4) count with pregnancy status to determine ART eligibility so that all pregnant and breastfeeding women are on lifelong ART [3]. In addition to its cost-effectiveness, Option B+ has significant advantage of reducing the MTCT of HIV to less than 2 % and reduces the sexual transmission of HIV to uninfected partner [10, 11]. ART used by HIV positive mothers during and after pregnancies averted an estimated 1.3 million new HIV infection among children for the last 5 years [7].

In 2011, Ethiopia adopted Option A, as a strategy of reducing MTCT of HIV, which used CD4 count and clinical stage as criteria for ART eligibility in pregnant women. Non-eligible women were recommended to take daily Zidovudine (AZT) starting from 14th week of gestation and continued during labor and for 7 days postpartum. However, low access to CD4 count test, low institutional delivery rate and loss-to-follow-up from care caused great challenges to the program. As a result, the strategic shift to Option B+ was endorsed in August 2012 and currently it’s being implemented in all health facilities providing PMTCT services [12, 13].

Although implantation of Option B+ enabled seven out of 10 pregnant women living with HIV to receive ART for PMTCT of HIV, MTCT rate is high up to 18% after breastfeeding ends. Despite 65% reduction in incidence of child HIV since 2009, there were 4800 new HIV infections among children in 2014 in Ethiopia [14]. Follow up studies done in Gondar, Jimma and Dire Dawa Hospitals’ PMTCT clinic showed 10 to 17% of infants born to HIV-infected mothers on PMTCT were HIV positive [15–17]. A cohort study conducted in Addis Ababa also showed that 8.4% of HIV exposed infants were HIV positive [18].

Reducing new pediatric HIV infections due to MTCT to less than 50 per 100,000 live births and a transmission rate of either less than 5 % in breastfeeding populations or less than 2 % in non-breastfeeding populations has been set by WHO as minimum impact target for elimination of MTCT of HIV [19]. In line with this, the Federal Ministry of Health of Ethiopia developed a three-year (2013–2015) plan of elimination of mother to child transmission of HIV, which aimed at providing ART for 90% of HIV positive pregnant women; ARV prophylaxis for 90% of HIV exposed children and reduce the vertical transmission of HIV to less than 5 % by 2015. However, as of June 2014, 12.8% of infants born to HIV positive mothers were HIV infected [20].

**Fig. 1:**
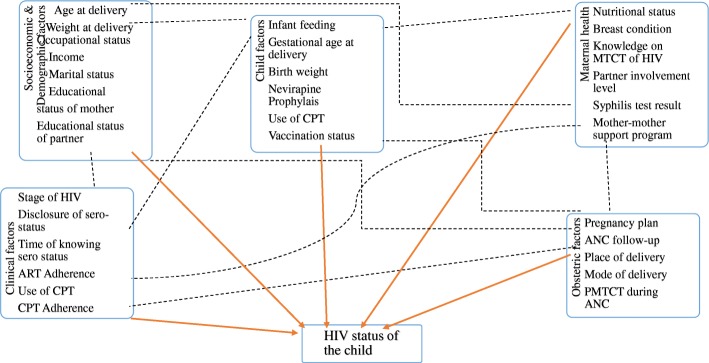
Conceptual framework describing determinants of HIV among children born to mothers on PMTCT: developed from review of related literatures [21–23]. Relation not assessed by this research; Investigated by this research

Literatures on the topic identified different sociodemographic, maternal, obstetric and child related factors determining MTCT of HIV during pregnancy, delivery and post-natal period (Fig. 1).

Identifying determinants of HIV infection among children helps understanding of the reason why transmission rate is high despite wide spread use of ART as per Option B+ and to regain achievements in child survival that HIV/AIDS have erased. Identifying factors associated with MTCT of HIV will have paramount importance in attaining United Nations AIDS target of eliminating new HIV infections among children [1]. Limited studies have described the determinants of HIV infection among children born to mothers on PMTCT program in Ethiopia; even those few studies used medical records that do not incorporate many more potential factors. Therefore, the aim of this study was to identify determinants of HIV infection among children born to mothers on PMTCT program of HIV in Addis Ababa. Thus, the findings of this study is expected to enhance evidence-based practices that improve PMTCT services and to realize the envisaged target of eliminating mother to child transmission of HIV.

## Methods

### Study area and period

The study was conducted in Addis Ababa city, Capital of Ethiopia. According to data obtained from Addis Ababa city health bureau, there were about 108 governmental health facilities including six regional hospitals, five federal hospitals and 97 health centers in Addis Ababa city and, all of them report to the city health bureau. All of them provide comprehensive maternal and child health care services, including ANC, PMTCT, safe delivery, postnatal care and counseling on infant feeding. There were 1673 HIV positive mothers who gave birth in those health facilities from January 1st, 2014- December 31st, 2015; and 67 of the children born to those mothers were found to have been positive for HIV within 24 months of follow-up period. This study was conducted from April to May, 2017 among 108 government health facilities, reporting to the city health bureau.

### Study design and population

Unmatched Case–control study design was conducted among HIV positive mothers who were on PMTCT program in Addis Ababa with their children who had confirmed HIV test results at or before 24 months of age.

A case was defined as HIV positive mother who had been on the PMTCT program with her child tested confirmed HIV positive at or before 24 months. A Control was HIV positive mother who had been on the PMTCT program with her child tested definitive HIV negative at 24 months. The HIV statuses of the mother, the partner and the child were obtained from their records.

### Exclusion criteria

Children accompanied by someone else other than their mothers or children whose mother were deceased or transferred-out to facilities outside of Addis Ababa were excluded from the study.

### Sample size

Sample size was calculated using Epi Info™ Version 7 StatCalc function of Sample Size Calculation for Unmatched Case-Control Study at 95% confidence interval (CI) and power of 80%, assuming 7 % of HIV negative children were exposed to mixed feeding and odds ratio (OR) of 3.55 based on similar previous study [21], which gave the largest sample size, considering 1: 4 ratio of cases to controls; thus, a sample size of 220 child-mother pairs (44 cases and 176 controls) was estimated.

### Sampling technique and procedures

Among all health facilities reporting to Addis Ababa city health bureau those who reported at least one HIV positive child born to mothers on PMCT program of HIV from 01 January, 2014 to 31 December, 2015 were selected purposively from Addis Ababa city health bureau annual reports. Accordingly, 22 health centers and four hospitals were included into the study.

**Fig. 2:**
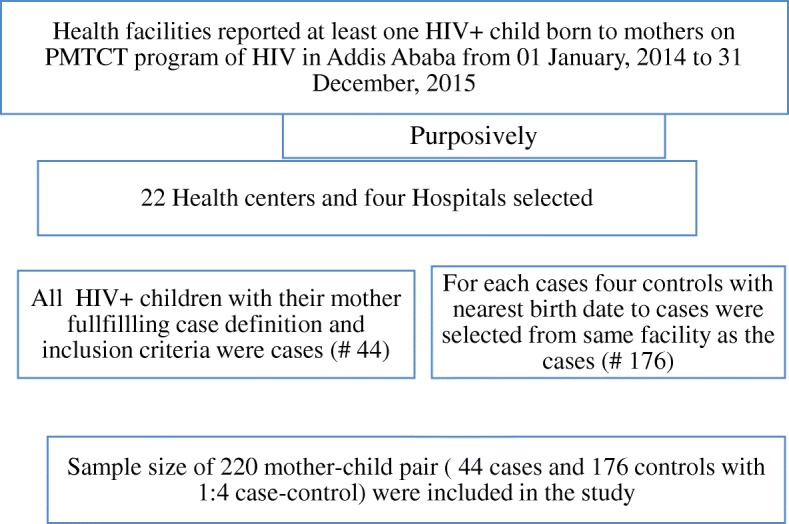
Sampling procedure for case control study on determinants of HIV infectionamong children born to mothers on PMTCT in Addis Ababa, 2017

Since, number of HIV positive children born to mothers on PMTCT program in government health facilities in Addis Ababa during the period from 01 January 2014 to 31 December 2015 were only 67 children, all of them who met the inclusion criteria were recruited into the study. Consequently, all the 44 HIV positive children with their mothers were considered as cases and for each case four controls with the nearest date of birth to the cases were recruited from the same health facilities as the cases (Fig. 2).

### Data collection techniques and procedures

The questionnaire was developed based on integrated PMTCT registration, exposed infants’ care follow-up records, medical record of the mother and the child. Additional potential variables were also incorporated and translated to local language (Amharic) and retranslated back to English to check for consistency and administered to the respondents with the Amharic version (Additional file 1).

Pretest of the tool was done in March, 2017 at Bishoftu Hospital, outside the study area that have similar setting with the study area, on 5 % of calculated sample size and appropriate modification was made. The data collected during pretest were not included in this report. In order to ensure confidentiality of their information, data were collected by trained and experienced clinical nurses and diploma midwifes who have at least 2 years of experience working at PMTCT clinic of the same facility.

Data related to CD4 count, viral load, WHO clinical stage, syphilis test results during pregnancy, birth weight, gestational age, duration of labor and rupture of membrane, adherence to ART and Cotrimoxazole prophylaxis therapy (CPT), sero status of the mother, the partner and the child were obtained from medical records. Whether pregnancy was planned, questions demonstrating partner involvement level and knowledge on MTCT of HIV were among variables collected by interviewing the mother. Some of these variables were cross checked against recorded values so as to reduce recall bias. Height and weight of the mothers were measured by nurses who were blinded for case or control status of the mothers.

### Operational definitions

#### Definitive HIV test results

HIV test result identified with DNA/PCR prior to 18 months of age, or by rapid antibody test after 18 months of age and 6 weeks of cessation of breast feeding [10].

#### Mothers on PMTCT

Mothers who have taken ART for prevention of MTCT of HIV either during pregnancy or child birth and delivery or during breastfeeding [10].

#### Nutritional status of the mother

Measured using Mid Upper Arm Circumference (MUAC): If > 22 cm = Not malnourished, ≤ 22 cm = Malnourished [10].

#### Knowledge about MTCT of HIV

Knowledge index was built using answers to six questions: three on possible periods of MTCT of HIV and three questions on possible ways of preventing MTCT of HIV; then, those who answered 60% and above of the questions was categorized as having high knowledge and < 60% was taken as having low knowledge [22, 23].

#### Partner

A person with whom the woman had intimate sexual relationship and became pregnant with the index child.

#### Partner involvement level

The level of partner involvement in PMTCT program was measured using six questions. A total score of four to six was considered as a ‘high’ partner involvement and less than four as ‘low’ partner involvement [24].

#### Participated in mother to mother support group

Mother member of formally organized group formed by HIV positive mothers who pass through PMTCT services and participated in at least one regular meeting.

#### ART adherence

Measured based on number of missed doses within 60 days. Three or less doses, four to eight doses, nine or more doses rated as good, fair and poor respectively [10].

#### CPT adherence

Measured based on number of missed doses per month. Less than three doses, three to nine doses, more than nine doses rated as good, fair and poor respectively [10].

### Data processing and analysis

Data were coded and double entered in to Epidata version 3.1 and exported to SPSS version 22 for cleaning and analysis. Exploration and cleaning of data were made to check for any inconsistencies, errors in coding, missing values, out of range values, unexpected data or outliers and inconsistencies were cross checked with the data in hard copy and necessary correction measures were taken.

The principal outcome variable was HIV status of the child coded as 1 for cases (HIV+) and 0 for controls (HIV-). Bivariate logistic regression analyses were performed to nominate candidate variables for multivariable analysis and those potential variables associated with the outcome at *P* ≤ 0.10 were included into the initial multiple logistic regression models, using backward fitting. Variables persisted to be associated with the outcome at *P* ≤ 0.05 were used in the final model. Adjusted Odds ratio (AOR) with its 95% CI was considered to judge for precision and decide whether independent association between outcome and independent variables exist.

Multicollinearity test was done to check whether independent variables were intercorrelated using variance inflation factor (VIF) and value of ten or more was considered for diagnosing multicollinearity and there were no seriously correlated variables. Reliability of the tools was measured using Cronbach’s alpha value and scales with value above 0.74 was accepted as reliable.

The log likelihood ratio test was used to test overall model fitness, measured based on statistical significance of the model chi-square displayed under Omnibus test of model coefficients and probability of model chi-square less than 0.05 supported the model to be good fit. In addition, Hosmer and Lemeshow test goodness of fit statistic value of more than 0.05 were used to characterize a logistic regression model as better fit.

A Wald test was used to test the statistical significance of relationship between MTCT of HIV and individual independent variables in the model. If the probability of the Wald statistic for the variable was less than the level of significance of 0.05, the null hypothesis that β coefficient for that variable equal to zero was rejected and supports the relationship.

Comparability of cases and controls on some of the continuous sociodemographic characteristics was checked using independent sample T-test and significance level greater than 0.05 and confidence interval of mean difference including zero was used to accept the null hypothesis and decide no difference. Equality of variances assumption of the independent sample T-test was checked by Levene’s test and significance level greater than 0.05 was used to decide equality of variance.

### Data quality assurance

The questionnaire was designed based on integrated PMTCT register, exposed infant follow-up registration book, medical record and after extensive review of related journal articles. The questionnaire was translated to local language and administered with the Amharic version. Experienced data collectors and supervisors were recruited and training was given, including practices during the pretest. Close supervision was made and filled formats and questionnaires were checked on daily basis for completeness and consistency.

Data documentation sheet or code book was prepared and used to make data entry form in Epidata and additionally the CHK commands were applied in order to restrict out of range and illegal values. A new composite variable derived from four variables (region number, facility type, facility code and patient assigned number) was used as a unique identifier in order to prevent duplicate data entry. Data were also double entered, cross checked, validated and all the discrepancies were resolved.

## Results

### Sociodemographic characteristics

A total of 220 mother-child pair were included in the study. The study participants (44 cases and 176 controls) were compared on basis of their sociodemographic characteristics. Independent sample T-test supported that there was no statistically significant difference among cases and controls with respect to their demographic characteristics such as age at delivery (mean difference = − 0.38; 95% CI: -2.21, 1.45), weight at delivery (mean difference = 0.75; 95% CI: -0.31, 1.81) and height of the mother (mean difference = 1.37; 95% CI: -0.68, 3.42) (Table 1).

**Table 1:** Independent samples T- test displaying no difference between cases and controls on sociodemographic characteristics

	Levene’s test for equality of variances		T-test for equality of means					
	F	Sig.	T	Sig.	Mean difference	SE difference	95% CI	
							Lower	Upper
Age at delivery	0.62	0.43	−0.41	0.68	−0.38	0.93	−2.21	1.45
Weight at delivery	0.10	0.75	1.39	0.17	0.75	0.54	−0.31	1.81
Height of mother	0.12	0.73	1.32	0.19	1.37	1.04	−0.68	3.42

### Maternal factors

More than three fourth (76.7%) of controls and nearly half (47.7%) of cases had good knowledge regarding MTCT of HIV. More than half (52.8%) of controls and less than 20 % (18.2%) of cases have had high partner involvement level. More than 27% of cases and less than 3 % (6.3%) of controls had breast diseases like cracked nipple, fissure or breast abscess. Approximately five and 4 % of cases and controls were sero-discordant.

### Obstetric factors

Concerning ANC attendance 94.3% of controls and 77.3% cases attend ANC at least once and 97.2% of controls and 79.5% cases took PMTCT prophylaxis during ANC. Regarding timing of knowing sero status more than three in five (61.9%) controls and more than half (54.5%) of cases were newly diagnosed during their current pregnancy for the first time. Among those newly diagnosed most (93.6%) of controls and 62.5% of cases were enrolled to PMTCT care during ANC. Majority (85.1%) of known HIV positive mothers who didn’t transmit HIV to their children were on ART before entry to PMTCT and 80% of them who transmit HIV to their children were not on ART.

### Child related factors

The mean (standard deviation, SD) of birth weight of the child was 2424.1 (SD:155.0) grams for cases and 2455.4 (SD: 272.0) grams for controls. There was no statistically significant difference between cases and controls in terms of their birth weight as indicated by the independent sample T-test (mean difference: 31.3; 95% CI: -52.9, 115.6). More than three-fourth (75.6%) of controls and 18.2% of cases were exclusively breast fed with in the first 6 months of their life. Three-fourth (75%) of cases and 5.7% of controls were mixed fed during the first 6 months of their life. Half of controls and 11.4% of cases were fully vaccinated.

### Factors associated with MTCT of HIV

Controlling for possible confounding factors, mothers who were not participated in mother to mother support program were more than five times more likely to transmit HIV to their children compared to those who participated in mother to mother support program (AOR: 5.1; 95% CI: 1.4, 18.1). Mothers who had low partner involvement level were nearly seven times more likely to transmit HIV to their children compared to those who had high partner involvement level (AOR: 6.9; 95% CI: 1.4, 13.4). Mothers enrolled to PMTCT care during WHO clinical stage-II of HIV were more than three times more likely to transmit HIV to their children compared to mother who enrolled to PMTCT care at stage- I (AOR: 3.3; 95% CI: 1.2, 9.1). Mothers who poorly adhered to ART were more than three times more likely to transmit HIV to their children compared to those who had good ART adherence (AOR: 3.1; 95% CI: 1.3, 7.5).

Controlling for other factors, mothers who had positive syphilis test result during pregnancy were more than threefold more likely to transmit HIV to their children compared to those who were syphilis negative (AOR: 3.2; 95% CI: 1.2, 8.6). Compared to mothers who had normal breast during breast feeding those who had breast disease while lactating were more than four times more likely to transmit HIV to their children (AOR: 4.4; 95% CI: 1.9, 10.4). Mothers who were under nourished during 18th follow-up month were more than three times more likely to transmit HIV to their children compared to those who were not (AOR: 3.1; 95% CI: 1.4, 6.8). As compared to mothers who had planned pregnancy those who had unplanned pregnancy were more than tenfold more likely to transmit HIV to their children (AOR: 10.3; 95% CI: 3.9, 27.2). Mothers who did not take PMTCT prophylaxis during ANC were more than three times more likely to transmit HIV to their children compared to those who had taken PMTCT prophylaxis during ANC (AOR: 3.1; 95% CI: 1.3, 9.6).

Mothers who delivered at home were more than five times more likely to transmit HIV to their children compared to those who delivered at health institutions (AOR: 5.3; 95% CI: 1.4, 19.4). Comparing mothers who delivered by cesarean section those who gave birth by instrumental delivery were more than three times more likely to transmit HIV to their children (AOR: 3.3; 95% CI: 1.6, 7.2). Compared to children who exclusive breast fed those who took mixed feeding in the first 6 months of life were more than 12 times more likely to acquire HIV from their mother (AOR: 12.5; 95% CI: 2.9, 52.7).

On the other hand, educational status of the mother and the partner, disclosure of HIV status to partner, nutritional status of the mother at time of data collection, ANC visit, ART prophylaxis during labor, oral disease of the child during breast feeding and vaccination status of the child were included in the initial multivariate logistic regression model but they were statistically not significant and not displayed in the final model (Table 2).

**Table 2:** Factors associated to MTCT of HIV in Addis Ababa, Ethiopia, April 2017

Characteristics	Categories	Cases	Controls	COR [95% CI]	AOR [95% CI]	*P*-value
Mother to mother support	No	41	93	12.1 [3.6, 40.8]	5.1 [1.4, 18.1]**	0.000
	Yes	3	83	1.00		
Partner involvement	High	8	93	1.00		
	Low	36	83	5.0 [2.2, 11.5]	6.9 [1.4, 13.4]**	0.000
WHO Stage at enrollment	Stage I	20	160	1.00		
	Stage II	24	16	12 [5.5, 26.3]	3.3 [1.2, 9.1]**	0.019
ART adherence	Good	21	136	1.00		
	Poor	23	40	3.7 [1.8, 7.4]	3.1 [1.3, 7.5]**	0.011
Syphilis test result	Negative	31	163	1.00		
	Positive	13	13	5.3 [2.2, 12.4]	**3.2 [1.2, 8.6]**	0.000
Breast condition	Normal	32	165	1.00		0.000
	Breast disease	12	11	5.6 [2.3, 13.9]	4.4 [1.9, 10.4]**	0.001
Nutritional status at 18th month	Not malnourished	12	136	1.00		
	Malnourished	32	42	8.5 [4.0, 17.9]	3.1 [1.4, 6.8]**	0.000
Pregnancy planned	Yes	9	147	1.00		
	No	35	29	19.7 [8.6, 45.4]	10.3 [3.9, 27.2]**	0.000
PMTCT prophylaxis ANC	Yes	35	171	1.00		
	No	9	5	8.7 [2.7, 27.8]	3.1 [1.3, 9.6]**	0.008
Place of delivery	Institution	28	170	1.00		
	Home	16	6	16.2 [5.8, 44.8]	5.3 [1.4, 19.4]**	0.001
Mode of delivery	Vaginal	18	118	0.7 [0.3, 1.5]	1.8 [0.7, 4.7]	0.257
	Cesarean	11	48	1.00		
	Instrumental	15	10	6.5 [2.3, 18.4]	3.3 [1.6, 7.2]**	0.002
Infant feeding practice within 6 months	Exclusive breast	8	133	1.00		
	Mixed feeding	33	10	54.8 [20.0, 149.8]	12.5 [2.9, 52.7]**	0.000
	Exclusive replacement	3	33	1.51 [0.3, 6.0]	1.2 [0.20, 8.1]	0.072

**significant at *p* value < 0.05

## Discussion

This study analyzed maternal socio-demographic and clinical characteristics, obstetric factors, and infant factors associated with MTCT of HIV during pregnancy, at labour, delivery and during postnatal period via breast feeding.

Findings of this study show that lack of participation in mother to mother support program were significantly associated with MTCT of HIV because group discussion among peer support network and healthcare provider help understanding of PMTCT interventions like appropriate infant feeding options, acceptability and utilization of family planning methods and enhances decision-making ability. In addition, participating in support program help for subsiding related stigma and facilitates disclosure of sero-status which in turn would help in better adherence to ART leading to less viral load and reduced likely of transmitting the virus to the child [25].

Low partner involvement level was found to be independent predictor of MTCT of HIV in this study. This is similar to other previous studies carried out in the study area which might be due to low uptake of PMTCT interventions related to low level of partner involvement as men plays an important role deciding whether the women has to go to health facilities for ANC and delivery, choosing safe infant feeding option and affects ART adherence [23].

Advanced WHO clinical stage of HIV at enrollment to PMTCT significantly increases MTCT of HIV as supported by related studies done in Ethiopia, particularly in Jimma [16] and Woliso [26]. Possible justification for this might be advanced stage of HIV indicated more opportunistic infections leading to high viral load and immunodeficiency. Another significant finding of this study was the association between poor adherence to ART and MTCT of HIV. This finding is consistent with related study done in Kenya [27], which might be explained by reduction in viral load caused by good ART adherence.

Mothers who were infected by syphilis during pregnancy showed statistically significant positive association with MTCT of HIV, which might be attributed to genital ulcer caused by syphilis facilitates transmission of the virus as described by related study done in Ethiopia [28]. Breast infection of the lactating mothers also showed significant association with MTCT of HIV, which is consistent with comparable a similar study [21]. The possible explanation for this could be due to increased exposure to the viral particles caused by breast diseases like cracked nipple and fissure. Similarly, maternal undernutrition was found to increase the log odds of having an HIV positive child, which is also in agreement with a former study [29] done in resource limited setting. This could be due to the fact that undernutrition during pregnancy increases mothers’ susceptibility to various infections, and hence increased chance of HIV transmission to the newborns.

Again, unplanned pregnancy was found to increase the risk of MTCT of HIV which was also consistent with another related study done in South Africa [30]. Possible explanation for this might be women with unplanned pregnancy might not have recognized their pregnancy until later in gestation and might be reluctant to timely care seeking due to delayed decision whether or not to continue with the pregnancy. Absence of PMTCT prophylaxis during ANC was also significantly associated with MTCT of HIV, which is analogous to a related study done in north west Ethiopia [15]. This might be due to the fact that lack of prophylaxis increases the viral load and its chance to be transmitted from those mothers to their newborns.

The finding of this study showed that home delivery increases the chance of HIV transmission from positive mothers to their newborns and this is in line with the study done in Western Kenya. [27]. The rationale behind such fact could be mothers who delivered at home missed the opportunities of using ARV prophylaxes given to the mother during labor and to the newborn right after birth. In addition, those mothers who delivered at home are more likely not to attend ANC during their pregnancy and also miss the opportunity of taking PMTCT prophylaxis during ANC.

Infant feeding option in the first 6 months of life is important determinant for MTCT of HIV; thus, those infants who nursed with mixed feeding during this period was positively and significantly associated with MTCT of HIV. This finding is similar with other related studies done in Ethiopia [21] and in Zimbabwe [31]. The possible explanation could be irritation of infant’s immature gastrointestinal tract caused by additional foods which might facilitate entry of HIV viral particles from the mother’s breast milk to the blood stream.

Occupational status of the mother and maternal age at delivery period were not significantly associated with MTCT of HIV which is consistent with the findings of comparable case-control study done in Assela, Adama and Bishoftu Hospitals [21]. Similarly, knowledge about MTCT of HIV was not significantly associated with MTCT of HIV which is in contrast with the study done in Ghana [32]. This might have resulted from knowledge about MTCT of HIV might have been gained after the child had been infected with HIV or might be as a result of difference in study setting and time.

Some of the variables obtained from records like maternal viral load, initial CD4 count when enrolled to PMTCT care, duration of ART, types of prophylaxis received, gestational age at delivery, duration of labor, duration of rupture of membrane and nutritional status of the child during follow-up period were incomplete and hence were not included in the analysis.

## Conclusion

Different maternal, obstetric and child related factors determining MTCT of HIV during pregnancy, delivery and post-natal period were identified. Lack of participation in mother to mother support program, low level of partner involvement, maternal undernutrition, poor ART adherence level, advanced WHO clinical stage of HIV, breast disease while lactating, syphilis infection, unplanned pregnancy, lack of PMTCT prophylaxis during ANC, instrumental and home deliveries, and mixed infant feeding practice during the first 6 months of life are independent factors associated with MTCT of HIV.

Improving record keeping at health institutions is needed to truck factors affecting mother –to- child transmission of HIV. Further follow-up study including variables incomplete in records such duration of labor, types of prophylaxis received, duration of rupture of membrane, gestational age at delivery, maternal viral load, initial CD4 count when enrolled to PMTCT care and duration of ART can improve understanding of influential factors affecting mother –to- child transmission of HIV.

## Additional file

Additional file 1: English version questionnaire. (DOCX 32 kb)

## Abbreviations

AOR: Adjusted odds ratio
ART: Antiretroviral therapy
ARV: Antiretroviral
AZT: Zidovudine
BMI: Body mass index
CD4: Cluster of differentiation
CI: Confidence interval
HIV: Human immunodeficiency virus
IRB: Institutional review board
MTCT: Mother to child transmission
MUAC: Mid upper arm circumference
PMTCT: Prevention of mother to child transmission (of HIV)
SD: Standard deviation
SE: Standard error
SSA: Sub- Saharan Africa
VIF: Variance inflation factor
WHO: World Health Organization
