# Supplementary material for: Determinants of HIV infection among children born to mothers on prevention of mother to child transmission program of HIV in Addis Ababa, Ethiopia: a case control study
Source: BMC Infect Dis. 2018 Jul 13;18:327. doi: 10.1186/s12879-018-3217-3 (PMC6045847; doi:10.1186/s12879-018-3217-3)
Supplement: Supplementary file 1 — English version questionnaire. (DOCX 32 kb) [file 12879_2018_3217_MOESM1_ESM.docx]

# **ANNEX. I: ENGLISH VERSION QUESTIONNAIRE**

QUESTIONNAIRE FOR THE STUDY ON DETERMINANTS OF HIV INFECTION AMONG CHILDREN BORN TO MOTHERS ON PMTCT PROGRAM IN ADDIS ABABA, ETHIOPIA, 2017

Region number: _______

Facility type: _______

Name of Health Institution: ___________________

Health Institution code: _______

Cohort year: _______

Registration. no: ______

| **Q#** | **Questions** | **Response category** | **Remark** |
| --- | --- | --- | --- |
| **Part. I. Socio-economic and demographic characteristics** | | | |
| 101 | What was your age at delivery in years? | __________ | Check record |
| 102 | What was your weight at delivery in kg? | __________ | Check record |
| 103 | What is your Occupation? | 1. Housewife 2. Employee 3. Student 4. Trader 5. Daily laborer 6. Farmer 7. Others (Specify)__________ |  |
| 104 | How much is the monthly income of the family in Ethiopian Birr? | ________ |  |
| 105 | What is your marital status when you were pregnant? | 1. Single 2. Married 3. Widowed 4. Divorced 5. Separated |  |
| 106 | What is your educational status? | 1. Can’t read & write 2. Read and write 3. Grade 1-4 4. Grade 5-8 5. Grade 9-10 6. College and above |  |
| 107 | What is your partner’s educational status | 1. Can’t read & write 2. Read and write 3. Grade 1-4 4. Grade 5-8 5. Grade 9-10 6. College and above |  |
| **Part. II. Nutritional status of the mother** | | | |
| 201 | Mid upper arm circumference (MUAC) of the mother at 6^th^ month follow up? | __________ | See record |
| 202 | MUAC of the mother at 12^th^ month follow up? | __________ | See record |
| 203 | MUAC of the mother at 18^th^ month follow up? | __________ | See record |
| 204 | What is Current MUAC of the mother? | __________ | Measure |
| 205 | What is Current weight of the mother in kg? | __________ | Measure |
| 206 | What is Current height of the mother in centimeter? | __________ | Measure |
| **Part. III: Maternal ARV and Obstetric factors** | | | |
| 301 | How many times ever pregnant? | __________ |  |
| 302 | How many times gave birth? | __________ |  |
| 303 | Was your pregnancy planned? | 1. Yes 2. No |  |
| 304 | Did you attend Antenatal care when you were pregnant? | 1. Yes 2. No | If No, Go to Qn 307 |
| 305 | How many Antenatal care visit(s) have you made? | _________ |  |
| 306 | Did you take PMTCT prophylaxis during ANC | 1. Yes 2. No | Check in record |
| 307 | What was mode of delivery? | 1. Emergency Cesarean section 2. Elective Cesarean section 3. Spontaneous vaginal delivery 4. Instrumental delivery 5. Episiotomy | Check in records |
| 308 | When did you knew your HIV sero status? | 1. Newly diagnosed during current pregnancy 2. Confirmed positive prior to current pregnancy | Check in records |
| 309 | What was your Syphilis test result during Antenatal care follow-up? | 1. Positive 2. Negative 3. Not done | See record |
| 310 | Date of delivery | ___________ | See record |
| 311 | Place of delivery | 1. Delivered at same facility 2. Delivered at another facility 3. Home delivery 4. Others(specify)__________ | Check in record |
| 312 | Duration of labor in hours | ________ | Check in record |
|  | Have you taken ART during labor? | 1. Yes 2. No | Check in record |
| **Part IV: Maternal clinical and immunological factors** | | | |
| 401 | Did you take PMTCT prophylaxis during ANC | 1. Yes 2. No |  |
| 403 | ART unique ID #? |  | See record |
| 404 | Were you part of mothers-mother support program and attend at least one regular meeting session? | 1. Yes 2. No |  |
| 405 | Did your partner attend Antenatal care with you? | 1. Yes 2. No |  |
| 406 | Did your partner knew your Antenatal care schedule? | 1. Yes 2. No |  |
| 407 | Did your partner supported your antenatal visits financially? | 1. Yes 2. No |  |
| 408 | Have you discussed antenatal interventions with your partner? | 1. Yes 2. No |  |
| 409 | Was your partner tested for HIV with you? | 1. Yes 2. No |  |
| 410 | Was your partner asked you if he could use condom when you were pregnant? | 1. Yes 2. No |  |
| 411 | Have you disclosed your HIV status to your partner? | 1. Yes 2. No |  |
| 412 | Could HIV be transmitted from an infected mother to her child? | 1. Yes 2. No | Go to Q 415 |
| 413 | What are the possible periods of MTCT of HIV (do not read the options)? | 1. During Pregnancy 2. During Labor and delivery 3. During Breast feeding |  |
| 414 | Can you mention three possible ways of preventing MTCT of HIV? | 1. Use of ART both by mothers and child 2. Safe obstetrical practice 3. Exclusive breast feeding |  |
| 415 | What was the partner’s sero status? | 1. Reactive 2. Not reactive 3. Not tested | Check in record |
| 416 | Initial CD4 count of the mother when enrolled to PMTCT? | ___________ | See record |
| 417 | WHO clinical stage of the mother when enrolled to PMTCT? | __________ | See record |
| 418 | Did you take Cotrimoxazole prophylaxis therapy (CPT)? | 1. Yes 2. No | Check in record |
| 419 | Mother's Breast condition; if breast feeding | 1. Normal 2. Cracked nipples 3. Mastitis 4. Breast Abscess 5. Other (specify): ________ | See record |
| 420 | ART adherence | 1. Good 2. Fair 3. Poor | See record |
| 421 | Cotrimoxazole Prophylaxis Adherence | 1. Good 2. Fair 3. Poor | See record |
| **Part. V: Infant Factors** | | | |
| 501 | Infant’s medical record number (MRN)? |  | See record |
| 502 | What was the birth weight of your child in grams? | __________ | See record |
| 503 | Gestational age at delivery in weeks? | __________ | See record |
| 504 | Did your child take Cotrimoxazole prophylaxis? | 1. Yes 2. No | Check in record |
| 505 | Did the infant receive Nevirapine prophylaxis during delivery? | 1. Yes 2. No | Check in record |
| 506 | Did the infant receive Nevirapine prophylaxis after delivery? | 1. Yes 2. No | Check in record |
| 507 | Infant feeding practice within the 1^st^ 6 months? | 1. Exclusive breast feeding 2. Exclusive replacement feeding 3. Mixed feeding | Check in record |
| 508 | Definitive sero status of the child? | 1. Positive 2. Negative 3. Unknown | See record |
| 509 | Did the child receive vaccination? | 1. Yes 2. No | Check in record  If No, Qn 511 |
| 510 | If yes to Qn 509 circle the types of the vaccine taken  (circle all that apply) | 1. Bacillus Calmette-Guérin 2. Oral Polio vaccine (0) (1) (2) (3) 3. Pentavalent (1) (2) (3) 4. Pneumococcal vaccine (1) (2) (3) 5. Rota vaccine (1) (2) 6. Measles 7. Vitamin A | See record |
| 511 | Abnormal findings or diagnosis that may suggest HIV infection  (circle all that apply) | 1. None 2. Generalized lymphadenopathy 3. Oral Candidiasis 4. Purulent ear discharge 5. Pneumonia or lower respiratory tract infections 6. Persistent diarrhea 7. Hepatosplenomegaly 8. Severe skin lesions 9. Persistent Fever 10. Other (specify) | See record |

**Thank you for your cooperation!**
